# Osimertinib as neoadjuvant therapy in a patient with stage IIIA non-small cell lung cancer: a case report

**DOI:** 10.1186/s13256-021-02748-y

**Published:** 2021-04-24

**Authors:** Caroline Y. Chen, Charlene M. Fares, Daniel Sanghoon Shin

**Affiliations:** 1 Department of Medicine, Division of Hematology/Oncology, University of California Los Angeles, UCLA Medical Center, 757 Westwood Plaza, Los Angeles, CA 90095 USA; 2 Department of Medicine, Division of Hematology/Oncology, University of California Los Angeles, Los Angeles, CA USA; 3 Division of Hematology/Oncology, VA Greater Los Angeles Healthcare System, Los Angeles, CA USA

**Keywords:** Non-small cell lung cancer, Tyrosine kinase inhibitors, Neoadjuvant, Case report

## Abstract

**Introduction:**

Tyrosine kinase inhibitors (TKI) targeting epidermal growth factor receptor (EGFR) are approved for use in metastatic non-small cell lung cancer (NSCLC).

**Case presentation:**

Here we present a case of a African American patient with stage IIIA NSCLC treated with osimertinib in the neoadjuvant setting with concurrent radiation, followed by resection. The patient remains disease-free 4 months after surgery.

**Conclusion:**

This case report suggests that osimertinib may be effective as neoadjuvant therapy in resectable stage III disease. Additionally, we provide a summary of previous case reports and ongoing clinical trials for neoadjuvant EGFR inhibition in stage III NSCLC patients.

## Introduction

Lung cancer is the second most common cancer in both males and females, and the leading cause of cancer-related death, with an estimated 228,150 new cases and 142,670 deaths in 2019.^1^ Surgery is the recommended treatment for early-stage (I–II) non-small cell lung cancer (NSCLC), whereas metastatic disease is managed with systemic treatments including chemotherapy, immunotherapy, and targeted therapies such as tyrosine kinase inhibitors (TKIs). The management of stage III lung cancer is highly individualized based on patient factors and guided by multidisciplinary input, generally including a combination of chemotherapy, radiation therapy, and possible resection if deemed operable.^2-3^ Tyrosine kinase inhibitors are not yet approved for stage III disease; however, several clinical trials with epidermal growth factor receptor (EGFR) inhibition in the neoadjuvant setting are ongoing (Table 1).

**Table 1:** Select ongoing clinical trials for epidermal growth factor receptor tyrosine kinase inhibitors in the neoadjuvant setting

Study title	Conditions	Interventions	ClinicalTrials.gov identifier
Neoadjuvant Erlotinib for Operable Stage II or IIIA NSCLC With EGFR Mutations	Stage II NSCLC, Stage IIIA NSCLC	Erlotinib	NCT01470716
Icotinib as Neoadjuvant Therapy in EGFR-mutant Stage IIIA-N2 Non-small Cell Lung Cancer	EGFR-positive Non-Small Cell Lung Cancer	Icotinib	NCT03749213
Osimertinib in Treating Participants With Stage I-IIIA EGFR-mutant Non-small Cell Lung Cancer Before Surgery	Stage I NSCLC Stage IA NSCLC, Stage IB NSCLC, Stage II NSCLC Stage IIA NSCLC Stage IIB NSCLC Stage IIIA NSCLC	Osimertinib	NCT03433469
Neoadjuvant Afatinib Therapy for Resectable Stage III EGFR Mutation-Positive Lung Adenocarcinoma	Resectable EGFR positive stage III NSCLC	Afatinib	NCT04201756
Neoadjuvant Gefitinib followed by Surgery and Gefitinib In Unresectable Stage III NSCLC With EGFR Mutations (NEGOTIATE)	Unresectable EGFR positive stage III NSCLC	Gefitinib	NCT02347839

*NSCLC* non-small cell lung cancer, *EGFR* epidermal growth factor receptor

The current US Food and Drug Administration (FDA)-approved indications for osimertinib are as first-line therapy for EGFR mutation-positive advanced NSCLC or as second-line therapy in T790M mutation-positive advanced NSCLC patients that progress on a first-line TKI.^4^ Several case reports and small early-phase trials have described the use of TKIs in the neoadjuvant setting for stage III NSCLC with afatinib, erlotinib (improved response rate but without survival benefit), and gefitinib (tumor reduction noted upon surgery, no increase in postoperative complications).^5-7^ Here we present the first case report, to our knowledge, of osimertinib in the neoadjuvant setting for stage III NSCLC.

## Case presentation

**Fig. 1:**
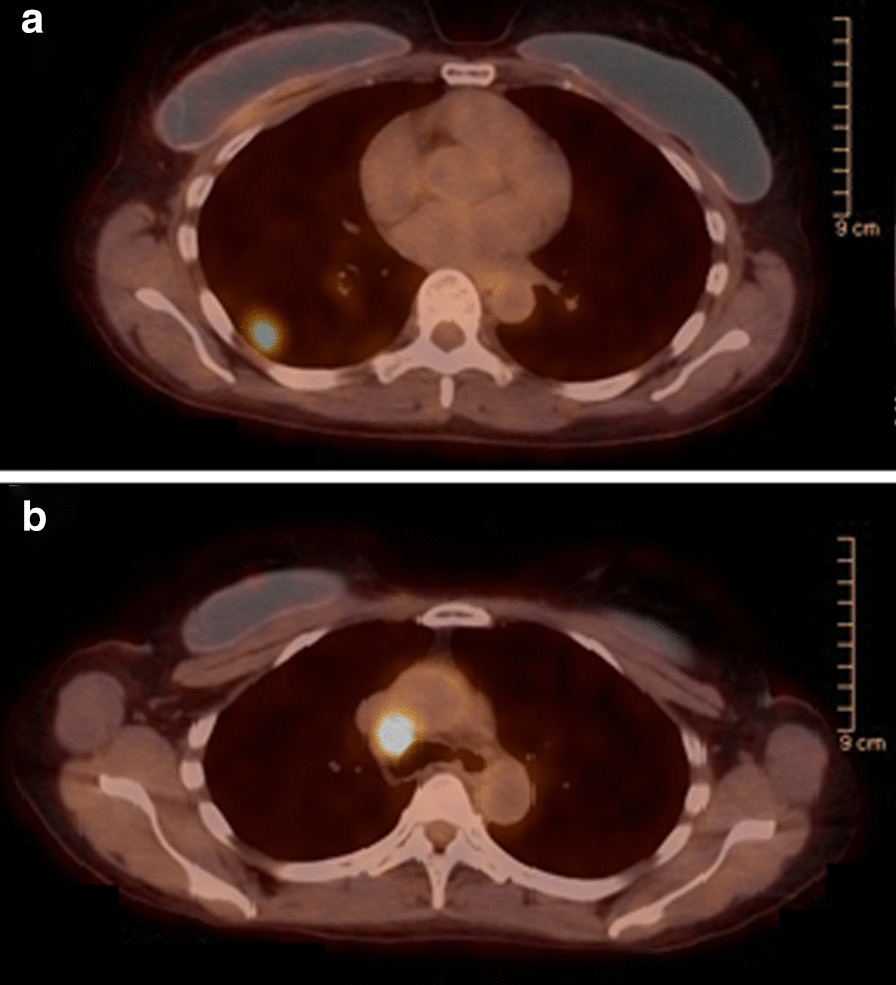
Disease status before and after neoadjuvant osimertinib therapy. Positron emission tomography/computed tomography at diagnosis. **a** Right lower lobe pulmonary nodule measuring 17 × 15 mm with maximum standardized uptake value (SUV_max_) of 7.6. **b** Prominent right paratracheal lymph node measuring up to 13 mm in the short axis with SUV_max_ of 12.9

A 64-year-old African American woman with no significant past medical history presented to the West Los Angeles Veterans Affairs (VA) Medical Center with tachycardia in August 2018. Chest X-ray showed a right lung mass, and follow-up computed tomography (CT) of the chest demonstrated a right lung nodular opacity 25 × 10 mm in size. Additionally, she was found to have mediastinal lymph node conglomerates approximately 15 mm in largest diameter, and right chest wall lymph nodes measuring 10 mm in the largest dimension. Endobronchial ultrasound (EBUS)-guided biopsy of the mediastinal lymph node demonstrated adenocarcinoma, with an EGFR mutation (exon 19 deletion). Positron emission tomography/CT (PET/CT) was remarkable for the same lesions previously visualized on chest CT which were fluorodeoxyglucose (FDG)-avid (Fig. 1a, b). Magnetic resonance imaging (MRI) of the brain revealed no evidence of central nervous system (CNS) involvement, making her malignancy consistent with stage IIIA (T1cN2M0) disease.

**Fig. 2:**
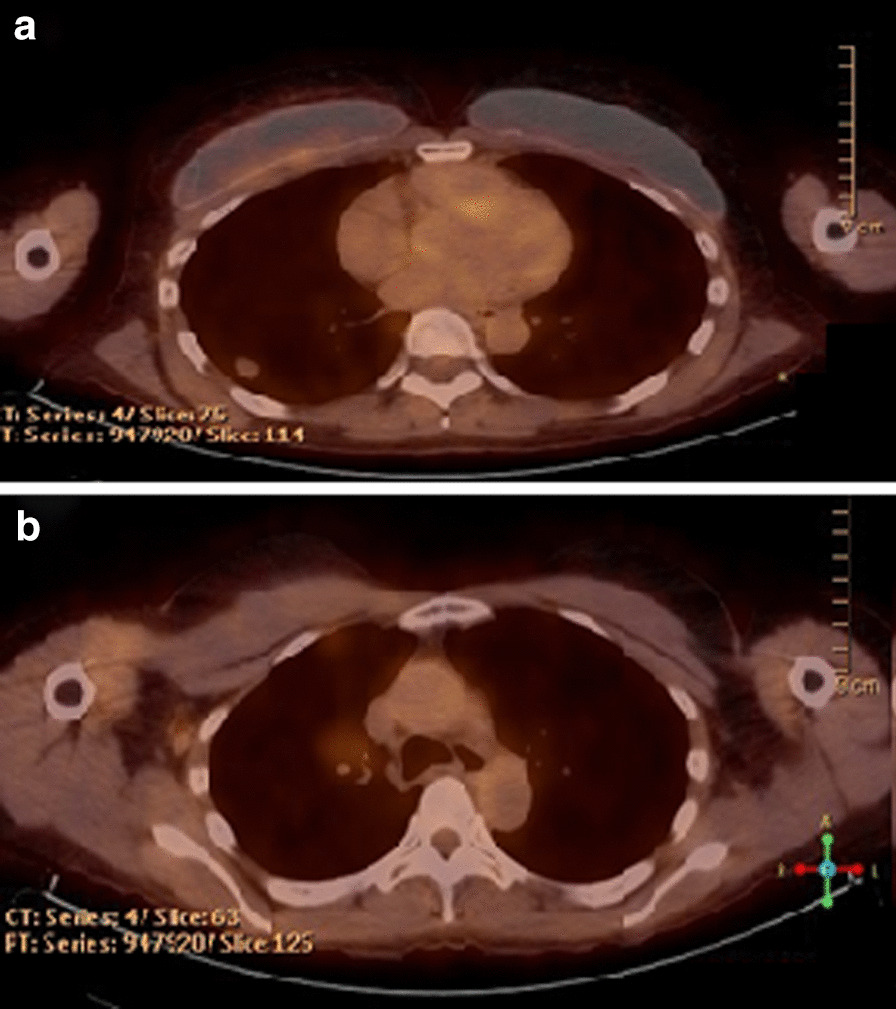
Disease status before and after neoadjuvant osimertinib therapy. Positron emission tomography/computed tomography 7 months after diagnosis and 2 months after treatment with neoadjuvant osimertinib. **a** Right lower lobe pulmonary nodule measuring 15 × 13 mm with maximum standardized uptake (SUV_max_) of 2.1. **b** Interval resolution of tracer activity of thoracic lymph nodes

**Fig. 3:**
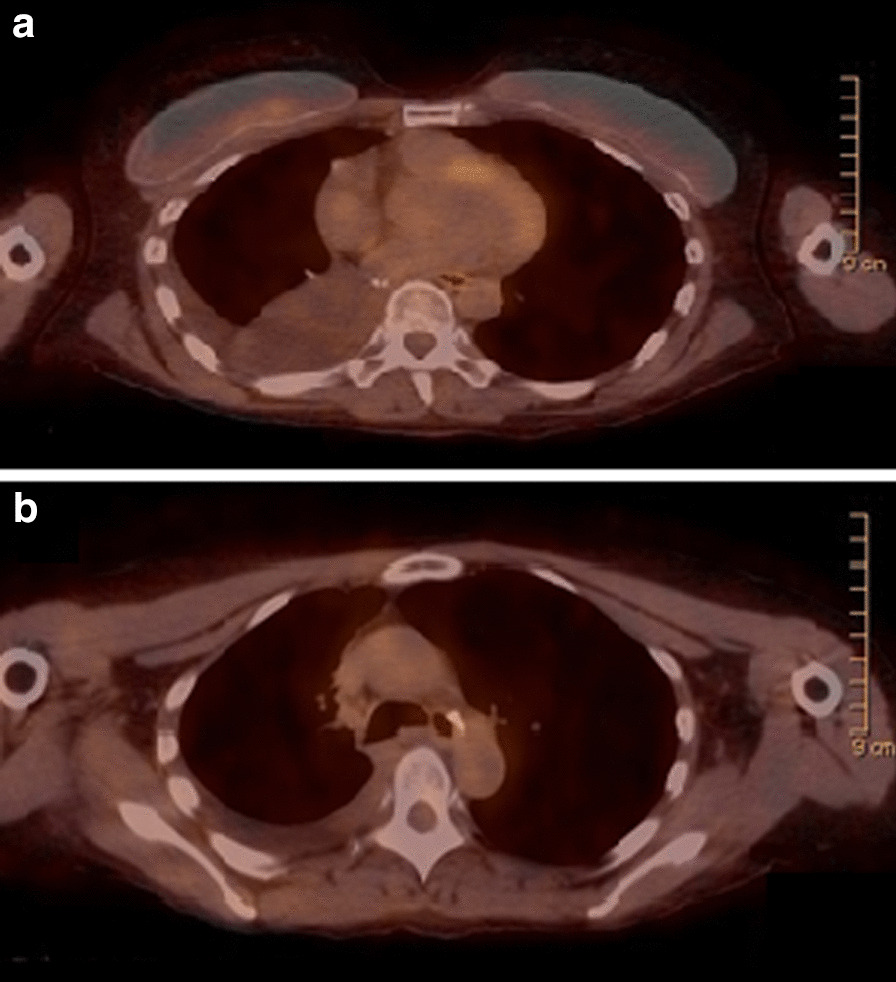
Disease status before and after neoadjuvant osimertinib therapy. **a**, **b** Positron emission tomography/computed tomography 10 months after diagnosis and 4 months after surgery. No evidence of disease

After multidisciplinary discussion at the tumor board, it was recommended that the patient undergo neoadjuvant treatment with chemotherapy and radiation, given N2 disease. The patient declined chemotherapy due to fear of side effects; however, she was amenable to immunotherapy or targeted therapy. Osimertinib 80 mg daily was eventually approved off label through the VA Pharmacy Benefits Management (PBM) program, which the patient began taking December 2018 for a total of 12 weeks of therapy, along with concurrent intensity-modulated radiation therapy (IMRT) with 200 cGy for 30 fractions, total of 6000 cGy (given during the last 6 weeks of osimertinib), which was tolerated well without adverse effects. PET/CT performed at 8 weeks into osimertinib therapy showed favorable treatment response, with a decrease in the right lower lobe lesion from 17 × 15 mm to 15 × 13 mm, decrease in FDG activity, and resolution of the 13 mm paratracheal lymph node initially seen on baseline PET/CT (Fig. 2a, b). Additional CT chest imaging was performed following completion of 12 weeks of osimertinib and 6 weeks of concurrent IMRT, which showed a further decrease in the size of the right lower lobe lesion to 12 mm in the largest dimension. Two weeks thereafter, the patient underwent robotic-assisted video-assisted thoracoscopic surgery (VATS) right lower lobe lobectomy, thoracic lymphadenectomy, which on pathology showed microscopic foci of residual adenocarcinoma spanning an area of 3 mm in the greatest dimension, without lymphovascular or visceral pleural invasion, 0/12 lymph nodes with tumor involvement. Most recent surveillance imaging with PET/CT approximately 10 months after presentation, and 4 months after surgical resection, showed no evidence of recurrent malignancy (Fig. 3a, b). Adjuvant therapy with osimertinib was discussed with the patient, who declined in favor of continued surveillance and re-initiation if her disease recurred.

## Discussion

Stage IIIA NSCLC, particularly in those with N2 disease, encompasses a heterogeneous group of patients that differ depending on extracapsular nodal involvement, single versus multilevel involvement, bulky versus non-bulky disease, and the location of the mass. Generally speaking, local management with surgery or radiation plus chemotherapy are largely equivalent with regard to overall survival and local control, and bimodal therapy (surgery or radiation plus chemotherapy) is equivalent to trimodal therapy (chemotherapy, radiation, and surgery). In contrast, patients with stage IIIB or higher NSCLC are managed with concurrent chemoradiation followed by up to 12 months of durvalumab.^2-3,8^

The use of targeted TKIs has revolutionized treatment of metastatic NSCLC in patients with EGFR-sensitizing mutations. However, evidence for use of these agents in earlier-stage disease is lacking, and efficacy in the neoadjuvant setting remains an important clinical question. Previous studies of EGFR inhibition in earlier stages have largely used gefitinib and erlotinib. Of note, the ADJUVANT/CTONG1104 and SELECT trials demonstrated improved disease-free survival after EGFR inhibition in the adjuvant setting with gefitinib and erlotinib, respectively. ^9-11^ Most recently, the ADAURA phase III clinical trial of osimertinib in the adjuvant setting for stage IB, II, or IIIA NSCLC demonstrated significantly improved 2-year disease-free survival of 90% versus 44% in favor of osimertinib.^12^ Further studies are needed to determine whether this holds true with EGFR inhibition in the neoadjuvant setting.

In this case report, the patient received osimertinib in combination with radiation therapy prior to surgery, after declining chemotherapy. Imaging obtained 8 weeks into therapy (reflective of 8 weeks of osimertinib therapy, and 2 weeks of concurrent IMRT) demonstrated partial response, and it is difficult to ascertain to what degree this response was due to osimertinib, IMRT, or the synergistic effect of both modalities. Nonetheless, she had a favorable response to treatment which allowed for complete resection of her tumor, and she remains disease-free 4 months following surgery. This case report suggests that neoadjuvant osimertinib is both effective and safe in resectable stage III NSCLC, and may be a viable option for patients who are not optimal chemotherapy candidates due to medical comorbidities, or for patients who decline chemotherapy.

Multiple questions remain regarding the use of EGFR TKIs in stage III disease. Optimal timing of neoadjuvant targeted therapy, radiation, and surgery remains unclear. In this case, the patient received concurrent radiation therapy which was tolerated well without added toxicity. This has been demonstrated in previous studies of TKIs (gefitinib, afatinib, and erlotinib) in combination with radiotherapy or chemoradiotherapy.^5-7^ Trials will be needed to assess whether combination with radiation therapy improves response when targeted treatments are used in the neoadjuvant setting. Additionally, few studies have examined neoadjuvant EGFR inhibition in unresectable stage III disease, with clinical trials currently ongoing to determine long-term outcomes. Finally, maintenance therapy with EGFR inhibition for patients who have demonstrated favorable response in the neoadjuvant setting should be considered.

## Conclusion

This is the first case report to show that osimertinib is effective in the neoadjuvant setting. Clinical trials are currently ongoing with neoadjuvant EGFR inhibitors for stage III NSCLC. This case report demonstrates proof of concept that neoadjuvant osimertinib may be effective for patients with resectable stage III NSCLC.

## Data Availability

Data sharing is not applicable to this article as no data sets were generated or analyzed during the current study.

## Abbreviations

TKI: Tyrosine kinase inhibitors
EGFR: Epidermal growth factor receptor
NSCLC: Non-small cell lung cancer
FDA: Food and Drug Administration
VA: Veterans Affairs
CT: Computed tomography
EBUS: Endobronchial ultrasound
PET/CT: Positron emission tomography/computed tomography
MRI: Magnetic resonance imaging
CNS: Central nervous system
PBM: Pharmacy Benefits Management
IMRT: Intensity-modulated radiation therapy
VATS: Video-assisted thoracoscopic surgery
